# Development of chloroplast simple sequence repeats (cpSSRs) for the intraspecific study of *Gracilaria tenuistipitata* (Gracilariales, Rhodophyta) from different populations

**DOI:** 10.1186/1756-0500-7-77

**Published:** 2014-02-04

**Authors:** Sze-Looi Song, Phaik-Eem Lim, Siew-Moi Phang, Weng-Wah Lee, Dang Diem Hong, Anchana Prathep

**Affiliations:** 1 Institute of Biological Sciences, University of Malaya, 50603 Kuala Lumpur, Malaysia; 2 Institute of Ocean and Earth Sciences (IOES), University of Malaya, 50603 Kuala Lumpur, Malaysia; 3 ACGT Laboratories, Lot L3-I-1, Enterprise 4, Technology Park Malaysia, Bukit Jalil, 57000 Kuala Lumpur, Malaysia; 4 Algal Biotechnology Department, Institute of Biotechnology (IBT), Vietnamese Academy of Science and Technology (VAST), Hanoi, Vietnam; 5 Seaweed and Seagrass Research Unit, Excellence Centre for Biodiversity of Peninsular Thailand, Department of Biology, Faculty of Science, Prince of Songkla University, HatYai, Songkhla 90112, Thailand

**Keywords:** cpSSRs, Genetic analysis, Haplotype, Rhodophyta

## Abstract

**Background:**

*Gracilaria tenuistipitata* is an agarophyte with substantial economic potential because of its high growth rate and tolerance to a wide range of environment factors. This red seaweed is intensively cultured in China for the production of agar and fodder for abalone. Microsatellite markers were developed from the chloroplast genome of *G. tenuistipitata* var. *liui* to differentiate *G. tenuistipitata* obtained from six different localities: four from Peninsular Malaysia, one from Thailand and one from Vietnam. Eighty *G. tenuistipitata* specimens were analyzed using eight simple sequence repeat (SSR) primer-pairs that we developed for polymerase chain reaction (PCR) amplification.

**Findings:**

Five mononucleotide primer-pairs and one trinucleotide primer-pair exhibited monomorphic alleles, whereas the other two primer-pairs separated the *G. tenuistipitata* specimens into two main clades. *G. tenuistipitata* from Thailand and Vietnam were grouped into one clade, and the populations from Batu Laut, Middle Banks and Kuah (Malaysia) were grouped into another clade. The combined dataset of these two primer-pairs separated *G. tenuistipitata* obtained from Kelantan, Malaysia from that obtained from other localities.

**Conclusions:**

Based on the variations in repeated nucleotides of microsatellite markers, our results suggested that the populations of *G. tenuistipitata* were distributed into two main geographical regions: (i) populations in the west coast of Peninsular Malaysia and (ii) populations facing the South China Sea. The correct identification of *G. tenuistipitata* strains with traits of high economic potential will be advantageous for the mass cultivation of seaweeds.

## Findings

### Background

*Gracilaria tenuistipitata* Chang and Xia is a red seaweed that is distributed not only throughout the tropical and subtropical regions in the Western Pacific [1], but also in the Indian Ocean [2]. This seaweed is cultured intensively in China for the production of agar and fodder for abalone [3,4]. This species’ wide tolerance to cultivation environment, high growth rate and high agar yield make it suitable for cultivation [5-7]. *G. tenuistipitata* was first described by Chang and Xia in 1976, and a new variety, *G. tenuistipitata* var. *liui*, was reported in 1988 [1]. *G. tenuistipitata* is found in Vietnam [8-10], Thailand [11], Philippines [12] as well as Kuah, Pulau Langkawi [2] and Batu Laut, Selangor [13,14] in Malaysia.

In seaweeds, the morphological species concept remains a basis for species-level and intraspecific studies [15]. However, seaweeds are well-known for their high morphological plasticity, and the same species of seaweeds can have a wide range of morphological features when grown under different environmental conditions. Correct identification is vital for commercial exploitation, such as with species of *Kappaphycus*, *Gracilaria*, *Sargassum*, and *Caulerpa*. Despite being chosen from the same clone and cultivated under identical conditions, these species may still vary in terms of yield and morphological features. Hence, molecular markers are pivotal for the observation and exploitation of genetic variations, which include genome structure, organization, and evolution throughout the entire genome [16]. Moreover, molecular markers provide an important tool for evaluating the levels and patterns of genetic diversity and have previously been utilized successfully in a variety of plant species [17-19].

Microsatellites, or simple sequence repeats (SSRs), are tandem repeats of 1–6 nucleotides that are distributed throughout the genomes of most eukaryotic species [18]. SSRs are the markers of choice for a variety of applications in plant genetics and breeding because of their multiallelic nature, higher levels of polymorphisms, codominant inheritance and relatively abundant [20]. Microsatellite loci have been successfully identified in a number of seaweeds, such as the red *Gracilariopsis lemaneiformis* [21], the green *Ulva intestinalis* [22] and the brown *Saccharina (Laminaria) japonica* [23]. The conventional method of developing SSR markers by generating genomic libraries after sequencing large numbers of clones to search for the SSR-containing DNA regions [24] is labor-intensive, time-consuming and costly [25-27]. An abundance of DNA sequence information has now been generated and deposited in online databases with the establishment of genome and expressed sequence tag (EST) sequencing projects in many algal species. These highly polymorphic SSR markers are useful in germplasm characterization, marker-assisted selection, cultivar identification, genetic diversity and phylogenetic relationship studies [28].

To date, the potential usage of microsatellite markers in seaweeds has been tested in *Gracilaria gracilis* [29] and *Porphyra haitanensis* [30] for assessing genetic variability, *Undaria pinnatifida* for exploring genetic structure [31], *Porphyra* sp. for germplasm identification [32], *Asparagopsis taxiformis* for determining ploidy level and sexual reproduction [33], and *Gracilariopsis lemaneiformis* [21] and *Chondrus crispus* for performing genetic diversity studies [34], among others. In Malaysia, molecular research has focused on *Gracilaria* species to investigate genetic relationships using random amplified polymorphic DNA (RAPD) [35,36] and genetic diversity using the mitochondrial *cox*1 gene [37]. cDNA libraries have also been generated for the Malaysian *Gracilaria changii* [38] and *Sargassum binderi* [39].

Chloroplast DNA (cpDNA) varies in terms of haploid nature, uniparental inheritance and lack of recombination compared with the nuclear genome [15,40]. The complete plastid genome sequences available in Rhodophyta include the chloroplast [41] and mitochondrial genomes [42] of *Porphyra purpurea* and the mitochondrial genome of *Chondrus crispus* [43]. In 2004, Hagopian *et al.* sequenced the chloroplast genome of *G. tenuistipitata* var. *liui*, which was the first plastid genome successfully completed from the subclass Florideophycidae (Rhodophyta) [44]. The life history of *G. tenuistipitata* var. *liui* was completed *in vitro* by Barufi *et al.* [45], and the mitochondria genome was successfully sequenced in 2010 [46]. Recently, expressed sequence tags of *G. tenuistipitata* var. *liui* were established and deposited in a public database, the National Center for Biotechnology Information (NCBI) [47]. This finding shows that there is increasing interest in *G. tenuistipitata* as a model organism for functional genomics studies.

Chloroplast SSRs (cpSSRs) have been used mostly in plant studies, such as the design of cpSSRs primers to amplify targeted regions in a diverse array of plant species [48], the development of universal primers to amplify SSRs in grasses by Provan *et al.* (2004a) [49] and also the development of a set of universal cpSSR primers to explore cpDNA diversity among sub-tropical and tropical fruit crops [50]. However, few studies of cpSSRs have been conducted in algae, such as the development of universal primers for the amplification of chloroplast coding and non-coding regions in Chlorophyta and Rhodophyta [51].

The aims of this study were to develop a set of microsatellite markers from the chloroplast genome of *G. tenuistipitata* using available bioinformatics tools and to test the derived primers on eighty specimens of *G. tenuistipitata* from six different localities to examine the genetic variation of *G. tenuistipitata* from different populations.

### Methods

#### Ethics statement

The Gracilaria tenuistipitata specimens were not collected from any national parks or protected areas. No specific permissions were required to collect the specimens.

#### Sample collection and DNA extraction

**Figure 1:**
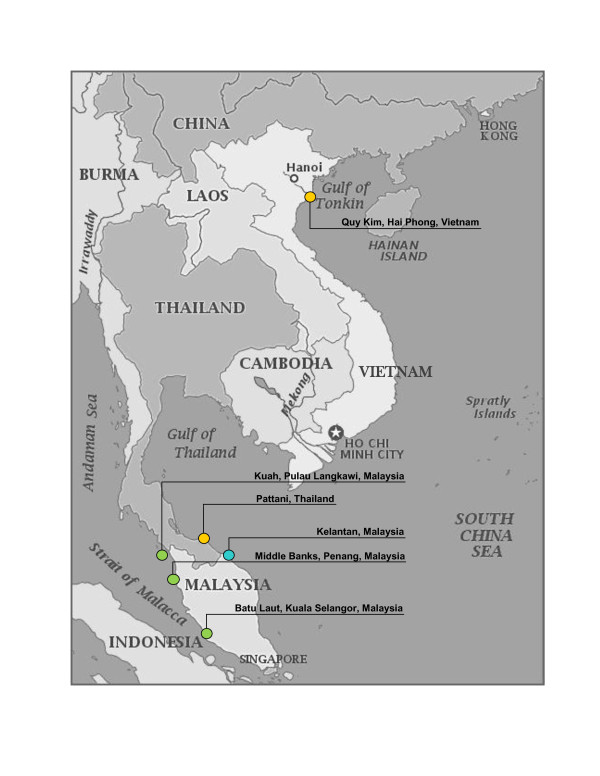
**Map displaying the six different locations where the *G. tenuistipitata *were collected.** The haplotypes of various localities are represented by different colors. Samples from Batu Laut, Middle Banks and Kuah (Malaysia) correspond to Clade A; Samples from Thailand and Vietnam correspond to Clade B1 and samples from Kelantan, Malaysia correspond to Clade B2. (adapted from http://www.yourchildlearns.com/online-atlas/Asia/vietnam-map.htm).

A total of 80 *Gracilaria tenuistipitata* specimens were collected from six different localities: i) Batu Laut, Selangor, Malaysia ii) Middle Banks, Penang, Malaysia iii) Kuah, Pulau Langkawi, Malaysia iv) Kelantan, Malaysia v) Quy Kim, Hai Phong, Vietnam and vi) Pattani, Thailand (Figure 1). Female gametophytes (haploid) and tetrasporophytes (diploid) were identified by their reproductive organs. The samples were grounded into powder in liquid nitrogen and DNA extraction was performed using the DNeasy Plant Mini Kit (Qiagen, Germany) according to the manufacturer’s instructions with minor modifications. The extracted genomic DNA was kept at -20°C for further analysis.

#### SSR development

The 183,883 bp *G. tenuistipitata* var. *liui* chloroplast genome DNA sequence was downloaded from GenBank (http://www.ncbi.nlm.nih.gov/) and saved in FASTA format. Perl scripts were developed to search for microsatellite tandem repeats in the genome sequence data using the MIcroSAtellite (MISA) search module (http://pgrc.Ipk-gatersleben.de/misa/). To avoid homoplasy, only perfect SSRs (with no substitutions interrupting the core motif) and nucleotides with repeated patterns of length two or more were used in this study*.* Primers were designed using Primer3 [52]. The parameters used for the design of SSRs were defined in such a way that the primer annealing temperatures varied from 48 to 55°C with primer length within 20–24 bp, GC content between 50 - 70% and the expected product size between 280 and 350 bp.

#### SSR analysis

Polymerase chain reactions (PCRs) were performed in a total volume of 15 μl containing 1.5 μl 10x PCR buffer (Takara Biotechnology, Dalian, China), 0.2 mM dNTP mix (Takara), 0.5 U *Taq* polymerase (Takara), 0.3 mM of each primer pairs, and 25–50 ng genomic DNA with UHQ water added to a total volume of 15 μl. The amplification reaction conditions were as follows: 5 min denaturation step at 94°C, followed by thirty-five cycles of 1 min at 94°C, 1 min at 52°C, 2 min elongation at 72°C, and a final extension at 72°C for 5 min. The forward primer of each primer pair was fluorescently [69-carboxy-fluorecine (FAM)] labelled. The optimized annealing temperature for PCR amplifications was 52°C and this optimum temperature was used to test all the specimens. The amplification products were separated on a 3.0% MetaPhor® agarose gel (FMC or Cambrex Corporation, USA) at 75 V for 75 min and visualized by SYBR SAFE staining (Invitrogen, USA). Amplification products were sent for fragment analysis to detect alleles using an automated DNA sequencer.

#### Data analysis

Primer-pairs that exhibited good amplification in all the eighty *G. tenuistipitata* samples were considered usable. SSR products were scored using a binary matrix method with “1” (presence) and “0” (absence) based on the SSR pattern that was amplified by each primer-pair designed. These DNA fingerprints of *G. tenuistipitata* specimens from different locations were constructed using GelQuest software [53] based on the size standard template ABI GeneScan 50–500 and followed with similarity matrix cluster analysis based on the UPGMA (unweighted pair group method using arithmetic means) using ClusterVis software version 1.4.2 [53].

The height of the peak greater than 50% the height of the highest peak was selected to avoid background noise, and the amplified fragment size analyzed was set in a range that included the expected amplified fragment size. Cluster analysis was performed based on the amplified fragment sizes of the samples, and the dendrogram was generated with the hyperbin width set to a value that will resulted in a group of defined amplified fragment sizes. The resulting UPGMA dendrogram was visualized and edited using the TreeMe software [54].

To verify that the polymorphisms were not because of indels in the regions flanking the cpSSRs, three PCR products that were randomly selected from each locality were purified and sequenced for each primer pair. The sequencing data were analyzed and edited using Chromas 1.45 (Technelysium Pty Ltd., Australia) and BioEdit 7.0.9.0 [55] software. Edited sequences were aligned by the CLUSTAL X program [56].

## Results

Eight perfect SSRs with designed primer-pairs (Table 1) were obtained from the 183,883 bp *Gracilaria tenuistipitata* var. *liui* chloroplast genome. Of the eight primer-pairs, five (62.5%) were mononucleotide repeats, two (25%) were dinucleotide repeats and only one (12.5%) was a trinucleotide repeat. All cpSSRs obtained were A, T or AT repeats. No CG microsatellites were identified. All primer-pairs were tested on 80 specimens of *G. tenuistipitata* from different localities. The eight primer-pairs demonstrated good amplification in 3% Metaphor agarose gel electrophoresis.

**Table 1:** **Primer pairs derived from the chloroplast genome of *G. tenuistipitata *** **var. *liui***

**Name**	**Forward primer (5′-3′)**	**Reverse primer (5′-3′)**	**Repeat motif**	**Predicted product size (bp)**	**Genomic coordinates of the SSR loci**	***Ta *** **(°C)**	**Coding/non-coding region**
GT1	TTTATCAACGATCCCTGTAG	AATGGACTGTAATTCACCAA	(T)_10_	336	4314 – 4323	52	Non-coding
GT2	TTTTTGAGCGATATTTTGAC	AGAATAAGACCACCTGAACC	(T)_10_	310	78590 – 78599	53	Coding
GT3	CCATAATGGAGATCTGTTTG	CTGGCAACATAGTTAGCATT	(T)_10_	306	84904 – 84913	53	Coding
GT4	AGCAATCCTAAATTGACAAC	AAGGTAGACCAGGAGAAAAA	(ATT)_5_	295	99538 – 99552	53	Coding
GT5	AGAAATTGATCAAGCTGTT	TTTTCAGCAATTGGAGTATC	(AT)_8_	329	105129 – 105144	52	Non-coding
GT6	CCTACAATCAGAATGGAATG	AGCTTCCAAGAAAAATGAGT	(A)_10_	303	143312 – 143321	53	Coding
GT7	ATCCTTCTTTTAAGCCGTAG	TCTTCCATGAAGTCTTCTTTT	(T)_10_	309	161988 – 161997	53	Non-coding
GT8	CTCCTGACATGATAAACACC	CTTCGATTTGTTTAATGAGC	(AT)_7_	288	179473 – 179486	52	Non-coding

*Ta* Annealing temperature.

Mononucleotide primer-pairs (GT1, GT2, GT3, GT6 and GT7) and the trinucleotide primer-pair (GT4) demonstrated good amplification but were monomorphic on the samples tested. Only one defined amplified fragment size, 336 bp for primer-pair GT1, 312 bp for primer-pair GT2, 308 bp for primer-pair GT3, 295 bp for primer-pair GT4, 301 bp for primer-pair GT6 and 307 bp for primer-pair GT7 was derived and the fragment results indicated that all samples fell within the same defined amplified fragment size.

For the primer-pair GT5, three defined amplified fragment sizes, 327 bp, 329 bp and 333 bp, were derived and samples with similar base size peaks fell into the same defined amplified fragment size (Table 2). The dendrogram for primer-pair GT5 indicated that the *G. tenuistipitata* specimens were grouped into three main clades: (a) *G. tenuistipitata* from Kelantan, Malaysia (b) *G. tenuistipitata* from Quy Kim, Hai Phong, Vietnam and Pattani, Thailand and (c) *G. tenuistipitata* from Kuah, Batu Laut and Middle Banks (Malaysia). All three clades were supported with similarity coefficients of 0.5.

**Table 2:** **Alleles of each *G. tenuistipitata *** **from six different localities for primer-pairs GT5 and GT8**

**Location**	**Number of samples tested**	**Primer GT5**			**Primer GT8**	
		**Allele (327 bp)**	**Allele (329 bp)**	**Allele (333 bp)**	**Allele (284 bp)**	**Allele (294 bp)**
Batu Laut, Selangor, Malaysia	15	1	0	0	0	1
Middle Banks, Penang, Malaysia	15	1	0	0	0	1
Quy Kim, Hai Phong, Vietnam	15	0	1	0	1	0
Pattani, Thailand	15	0	1	0	1	0
Kelantan, Malaysia	10	0	0	1	1	0
Kuah, Pulau Langkawi, Malaysia	10	1	0	0	0	1

1, Presence of SSR product.

0, Absence of SSR product.

For the primer-pair GT8, two defined amplified fragment sizes, 284 bp and 294 bp were derived, and samples with similar amplified fragment sizes exhibited a cell value in that column. The dendrogram for primer-pair GT8 indicated that the *G. tenuistipitata* specimens were grouped into two main clades: (a) *G. tenuistipitata* from Batu Laut, Middle Banks and Kuah (Malaysia) and (b) *G. tenuistipitata* from Pattani (Thailand), Quy Kim, Hai Phong (Vietnam), and Kelantan (Malaysia). All two clades were also supported with similarity coefficients of 0.5.

**Figure 2:**
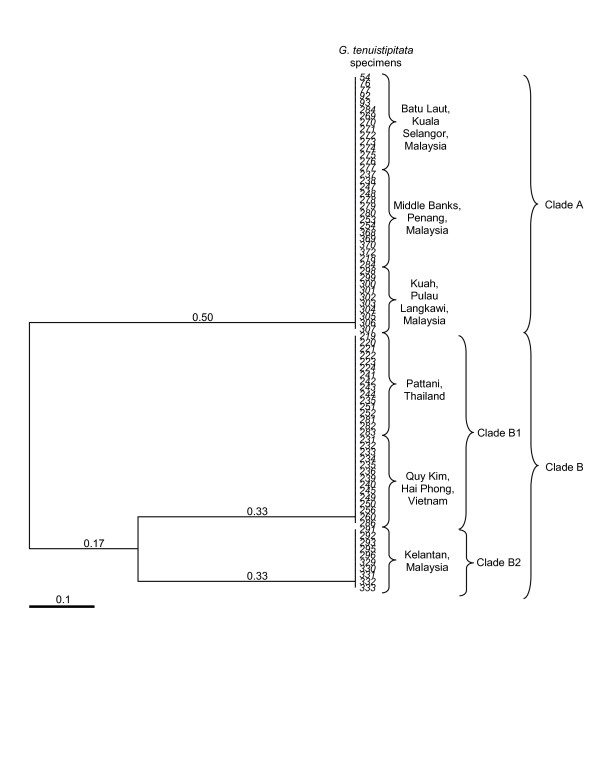
**Cluster analysis of eighty *G. tenuistipitata *** **specimens for the combined primer-pairs GT5 and GT8 from different localities using the unweighted pair group method with arithmetic average (UPGMA).**

The combined dataset from the two primer-pairs produced a dendrogram (Figure 2) with similarity coefficients ranging from 0.17 to 0.5. From the generated dendrogram, the 80 specimens of *G. tenuistipitata* were grouped into two major clades: Clade A consisting of *G. tenuistipitata* from Batu Laut, MiddleBanks and Kuah (Malaysia), and Clade B consisting of *G. tenuistipitata* from Pattani (Thailand), Quy Kim, Hai Phong, (Vietnam), and Kelantan (Malaysia). Clade B is further divided into two sub-clades: Clade B1 consisting of *G. tenuistipitata* from Pattani (Thailand) and Quy Kim, Hai Phong, (Vietnam), and Clade B2 consisting of *G. tenuistipitata* from Kelantan (Malaysia). Both clades B1 and B2 were supported with a similarity coefficient of 0.33.

The sequencing results indicated that the amplification products of these primer-pairs were generated because of polymorphisms and not indels in the regions flanking the cpSSRs (the sequences of the amplified products can be provided upon request).

### Discussion

In plants, the highest frequency of cpSSRs was 160 found in *Arabidopsis thaliana,* and the lower numbers of cpSSRs were found in *Nymphaea alba*, the water lilies with 35 and *Nuphar advena* with 39 [57]. Studies have suggested that the cpSSRs are abundant in most plants [58-60] but less abundant in algae. This finding is consistent with our analysis on SSRs identified from the 23 algae chloroplast genomes deposited at the National Center for Biotechnology Information database (data not shown here). The analysis indicated that approximately 50% of the chloroplast genome contains low numbers of repetitive sequences, particularly on SSRs. *Ectocarpus siliculosus, Parachlorella kessleri, Monomastix* sp*., Scenedesmus obliquus,* and *Chlorella vulgaris* were found to have one SSR; *Bryopsis hypnoides, Micromonas* sp*., Cyanidioschyzon merolae* and *Cyanidium caldarium* were found to have two SSRs; *Gracilaria tenuistipitata* var. *liui* and *Porphyra yezoensis* have three SSRs; and only *Leptosira terrestris* has more than 60 SSRs. These results demonstrate that *G. tenuistipitata* var. *liui* used in this study is in the category of low numbers of repetitive sequences.

Of the eight primer-pairs we designed, only two revealed genetic polymorphisms in the eighty specimens tested. Primer-pair GT5 differentiates the *G. tenuistipitata* into three main groups at the 0.5 similarity level by cluster analysis. This result demonstrated that the populations of *G. tenuistipitata* from Batu Laut, Middle Banks and Kuah that grow in the Straits of Malacca, on the west coast of Peninsular Malaysia, were separated from the populations of Thailand, Vietnam and Kelantan, Malaysia, which face the South China Sea (Figure 1).

Primer-pair GT8 was able to differentiate the populations of *G. tenuistipitata* from Vietnam and Thailand from the populations of *G. tenuistipitata* from Malaysia (Batu Laut, Middle Banks and Kuah), but not the population from Kelantan, Malaysia, as was achieved with the use of primer-pair GT5. Primer-pair GT5 indicated that there are three possible haplotypes of *G. tenuistipitata* from the samples collected, whereas primer-pair GT8 only indicated two haplotypes of *G. tenuistipitata.*

Using the combined dataset of the two primer-pairs (Figure 2), *G. tenuistipitata* from Kelantan, Malaysia was more closely related to the specimens from Quy Kim, Hai Phong (Vietnam) and Pattani (Thailand) and distantly separated from *G. tenuistipitata* from Batu Laut, Middle Banks and Kuah (Malaysia).

Two of the eight primer-pairs that were generated from the chloroplast genome of *G. tenuistipitata* were able to distinguish the populations of *G. tenuistipitata* from different geographical origins (west and east coast of Peninsular Malaysia) and the population from Kelantan, Malaysia was separated from other localities. The variation in the repeated nucleotides can be used not only in genetic mapping and marker-trait association studies [28,61] but also in genetic diversity and population studies. This information regarding genetic diversity and differentiation of strains of the same species is pivotal particularly in cultivation, where red algae have contributed to 24.5% of the total world seaweed production [62] for food, fodder, ingredients in cosmetics and fertilizers, and hydrocolloid production (e.g., agar and alginate) [63]. This study demonstrated that variation in SSR length and stability varies among loci within species [64,65], and microsatellites obtained from genomic libraries have been found to be more polymorphic [64,66]. However, whether are more polymorphic than ESTs of *G. tenuistipitata* must be proven by comparing SSRs developed from the two sources.

To obtain the optimal forward and reverse primers that flanked the SSR region and to ensure robust PCR amplification, such parameters as oligonucleotide melting temperature, primer size, GC content and PCR product size were designed to a defined set of constraints [67]. Our study indicated that the microsatellites mined from the chloroplast genome of *G. tenuistipitata* in both coding and non-coding regions are A/T rich, which also supports the findings that (AT)_n_ sequences are commonly found in genomic libraries of plants [68-70]. This result may be observed because of the excess of AT repeats and deficiencies of AC/TG and CG repeats. C or G mononucleotide repeats were rare or absent [57].

There is limited gene flow and a weak geographic structuring pattern, because the number of haplotypes was rather low. However, this can only be verified with wider samples from more populations. For example, the analysis of the distribution of genetic diversity of the seaweed *Chondrus crispus* by Provan and Maggs (2012) [34] included 19 populations from both sides of the North Atlantic using mitochondrial single nucleotide polymorphisms (SNPs), sequence data from two single copy nuclear regions and microsatellite loci. These researchers’ results revealed unique genetic variation for all marker classes in the rear-edge populations in Iberia but not in the rear-edge populations in North America. Thus, more samples from different populations and also different techniques of molecular assays will likely help us understand the migration and dispersal ability of the species.

### Conclusions

This study is consistent with the observation by Schaal *et al.* that the chloroplast genome is suitable for the design of simple sequence repeats because of its lower rate of mutation and therefore contains potentially more effective genetic markers of population subdivision and differentiation than the nuclear genome [71]. Indeed, there are a few studies of chloroplast markers in population genetic, biogeographic and hybridization [59,71-73]. Although the cpSSRs that we developed were not able to distinguish between different populations of seaweeds, the population from Kelantan, Malaysia was characterized by a specific haplotype and exhibited an interesting geographical pattern in which samples facing the Straits of Malacca have a different haplotype from those facing the South China Sea. It is important to choose and breed *G. tenuistipitata* with traits of high economic value and use them for cultivation. Additional studies on gel strength, gel contents and growth rates among the different populations of *G. tenuistipitata* will provide valuable information regarding strain selection for cultivation. Hence, molecular genetic studies, particularly on the development of molecular markers, are essential to effectively select such strains. Further studies concerning developing SSRs from the mitochondrial genome [46] and ESTs [47] of *G. tenuistipitata* may result in the development of molecular markers for differentiation between species (at the strain and population level) and among species, which will be important for aquaculture.

## Abbreviations

cpSSRs: Chloroplast simple sequence repeats; RAPD: Random amplified polymorphic DNA; SSRs: Simple sequence repeats; UPGMA: Unweighted pair group method with arithmetic average

## Competing interests

The authors declare that they have no competing interests.

## Authors’ contributions

PE and SM supervised the project. WW and SL mined the SSR primers. SL, PE, DH and AP provided the specimens. SL and PE analyzed the data and wrote the manuscript. All authors read and approved the final manuscript.
